# Recent Advances in Chlorogenic Acids for Food Preservation and Shelf-Life Extension

**DOI:** 10.3390/antiox15050633

**Published:** 2026-05-15

**Authors:** Dina Zhang, Fanqianhui Yu

**Affiliations:** 1 Haide College, Ocean University of China, Qingdao 266100, China; zhangdina@stu.ouc.edu.cn; 2 Department of Computer Science and Technology, Ocean University of China, Qingdao 266100, China

**Keywords:** chlorogenic acids, antioxidant, food products, encapsulation strategies, sensory evaluation

## Abstract

The use of antioxidants has become a fundamental approach in food preservation to mitigate the adverse effects of oxidative deterioration, such as lipid rancidity and protein degradation. As a result, chlorogenic acids (CGAs), natural phenolic compounds, have attracted considerable attention due to their potent antioxidant and antibacterial activity as well as their diverse bioactivities, which are primarily achieved through the direct scavenging of free radicals and indirect inhibition of signaling pathways. Based on this, this review introduces the various derivatives of CGAs and their numerous health benefits, such as hypotensive and hypoglycemic effects, anti-obesity activity, and gastrointestinal flora regulation, and discusses innovative added forms involving novel encapsulation methods such as microcapsules, nanocapsules, and hydrogels. Moreover, this paper also provides a comprehensive summary of the preservation effects and sensory evaluation of CGAs in the food field, which have been proven to significantly extend the shelf life and enhance antioxidant capability in seafood products, meat, and baked goods. Finally, it also highlights the practical limitations of CGAs, including their poor liposolubility, chemical instability, and high thermal sensitivity, as well as the need for their application in a wider range of foods and further research on their influence on sensory evaluation, in order to broaden their application as antioxidants in the future.

## 1. Introduction

Oxidation-induced food spoilage is one of the most important causes of food deterioration [1]. To extend shelf life and maintain food quality and safety, research on enhancing the preservation properties and sensory characteristics of food products using natural or synthetic antioxidants has gained increasing attention [2]. Among these, natural antioxidants are highly favored due to their natural origin and wide availability. They are a diverse group of bioactive metabolites [3], including phenolics, terpenoids, and vitamins [4]. They play a key role in providing diverse biological effects [5] and protecting cells from oxidative stress by suppressing the generation of free radicals, reactive oxygen species, and reactive nitrogen species [6].

As natural antioxidants, chlorogenic acids (CGAs) have attracted significant attention in recent years. CGAs are water-soluble, bioactive phenolic compounds found in sources such as coffee beans and potatoes. Their parent structure consists of the hydroxy group of quinic acid and the carboxyl group of caffeic acid, containing multiple phenolic hydroxyl groups, with the molecular formula $C_{16}H_{18}O_{9}$ [7]. Specifically, CGAs are widely distributed in various food wastes, such as tobacco waste [8,9,10,11], coffee solid waste [12,13], fennel waste [14], artichoke waste [15], potato waste [16], sunflower waste [17], and squash waste [18]. This indicates CGAs’ potential for reintegrating waste into the food system to promote eco-friendly and sustainable food processing. Moreover, CGAs stand out due to their multiple health benefits, including anti-inflammatory [19], immunomodulatory [20], anti-obesity [21], anti-diabetes mellitus [22], and neuroprotective activity [23]. The extraction methods of CGAs are characterized by high efficiency, time-saving capability and eco-friendliness. Apart from conventional solvent extraction using ethanol, methanol or acetonitrile [24], innovative techniques include microwave-assisted extraction [25], Fe-assisted hydrothermal extraction [8], and ultrasonic extraction [26].

At the same time, CGAs mainly rely on modulating signaling pathways such as mitogen-activated protein kinase (MAPK) and nuclear factor kappa-B (NF-κB) [27] to provide their strong antioxidant properties. In addition, they act as strong antibacterial agents by disrupting cell membrane integrity, inhibiting the key protein activity and blocking pro-inflammatory signaling pathways, which are all directly linked with food systems. The antioxidant activity of CGAs is primarily attributed to their phenolic structure, which can scavenge free radicals and inhibit lipid peroxidation effectively. CGAs exhibit 2–3 times the 2,2-Diphenyl-1-picrylhydrazyl (DPPH) radical scavenging capacity of vitamins C and E, and their superoxide anion radical scavenging activity is also 10–30 times greater [28,29]. Xin et al. [30] demonstrated that CGAs can inhibit radiation-induced apoptosis and DNA damage by scavenging excessive reactive oxygen species (ROS) and activating the NF-E2-related factor 2 (Nrf2) antioxidant system. More specifically, according to a study by He et al. [31], the addition of CGA significantly enhanced the antioxidant capacity of grass carp filets, extended their shelf life by 3 days, and increased the DPPH radical scavenging rate by approximately 60%. Moreover, CGAs exhibit strong antibacterial properties [32], further enhancing their application value in food preservation [33]. Kang et al. [34] demonstrated the combination of CGA and ultraviolet-A light (UVA, 365 nm) is effective in inhibiting *Escherichia coli* DH5α strains in an acidic environment, suggesting its potential application to a wealth of acidic food products. As expected, CGAs have found extensive applications in various sectors, such as pharmaceutical [35], food [36], healthcare [37], and chemical fields [38]. 

In addition, CGAs exhibit a favorable safety profile, showing no significant adverse effects or toxicity on normal cells or tissues and demonstrating good human tolerability. In a 90-day study, rats administered doses of 250, 500, and 1000 mg/kg of CGAs did not exhibit any adverse reactions [38]. Similarly, an acute toxicity experiment revealed no side effects in mice for 14 days upon intake of CGAs [39]. The European Food Safety Authority (EFSA) has issued scientific support for the labeling of antioxidant-related health claims for foods containing CGAs [40], with estimated dietary intake ranging from 5 to 1000 mg/d [41].

**Figure 1:**
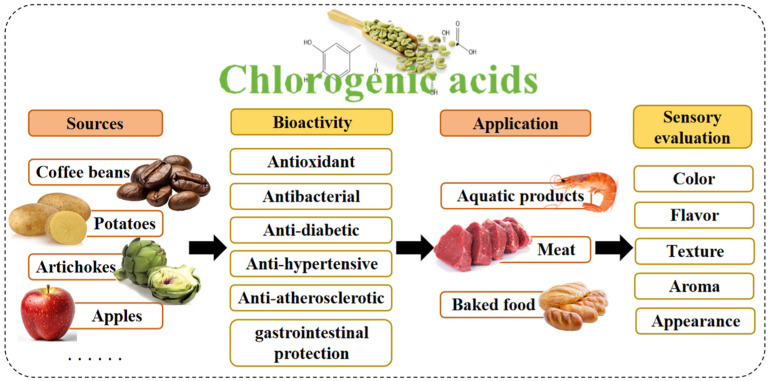
Introduction of CGAs.

Previous literature reviews have summarized the biological activities of CGAs on human health, but there remains a significant gap in guiding their application for food quality preservation and utilization. For instance, Lu et al. (2020) focused on the health benefits of CGAs and their mechanisms of action [42]. Li et al. (2023) focused on reviewing CGAs’ function as health agents and their applications in food packaging and quality protection, without systematically exploring their applications in different food products [43] and Nguyen et al. (2024) systematically reviewed CGAs’ pharmacological activity, medicinal properties, and mechanistic actions as potential therapeutic agents [44]. Based on this, this review aims to comprehensively outline the specific application forms of CGAs as antioxidants in food products and systematically summarize their effectiveness and research progress in various food preservation fields over the past five years, as shown in Figure 1. The information provided herein will guide the future application of CGAs as natural antioxidants in food preservation, where they hold significant potential to replace other natural antioxidants.

## 2. Properties of CGAs

### 2.1. Naturally Occurring Forms in Fruits and Vegetables

**Figure 2:**
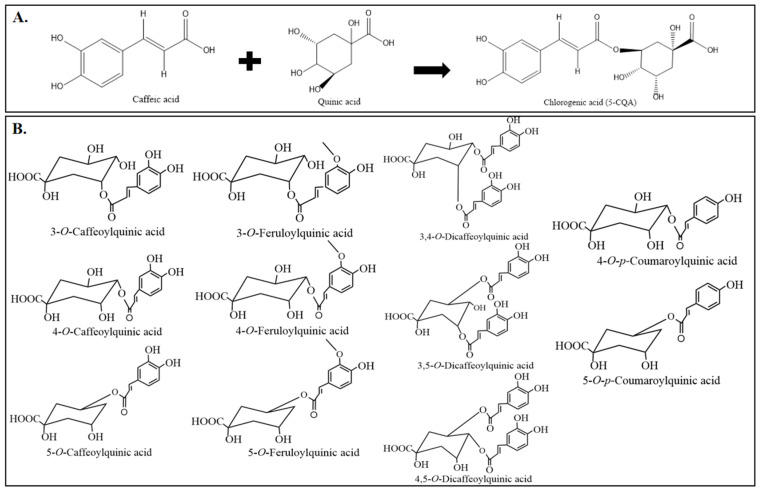
Chemical structure of CGAs. (**A**) General chemical structure of CGAs. (**B**) Structures of different isomers of CGAs.

**Figure 3:**
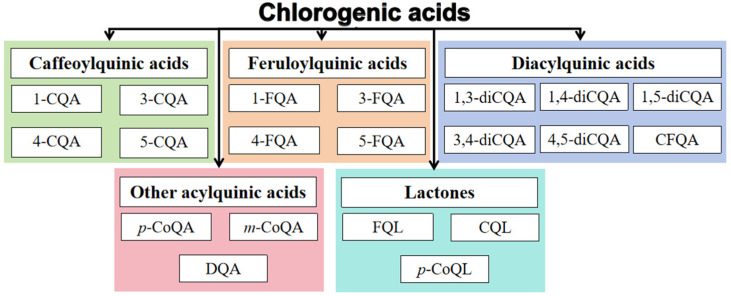
Different isomers of CGAs. Abbreviations: 1-CQA, 1-*O*-caffeoylquinic acid; 3-CQA, 3-*O*-caffeoylquinic acid, neochlorogenic acid; 4-CQA, 4-*O*-caffeoylquinic acid, cryptochlorogenic acid; 5-CQA, 5-*O*-caffeoylquinic acid, neochlorogenic acid; 1-FQA, 1-*O*-feruloylquinic acid; 3-FQA, 3-*O*-feruloylquinic acid; 4-FQA, 4-*O*-feruloylquinic acid; 5-FQA, 5-*O*-feruloylquinic acid; 1,3-diCQA, 3,5-dicafeoylquinic acid, isochlorogenic acid A; 1,4-diCQA, 1,4-dicafeoylquinic acid; 1,5-diCQA, 1,5-dicafeoylquinic acid; 3,4-diCQA, 3,4-dicafeoylquinic acid, isochlorogenic acid B; 4,5-diCQA, 4,5-dicafeoylquinic acid, isochlorogenic acid C; CFQA, caffeoylferuloylquinic acids; *p*-CoQA, *p*-coumaroylquinic acid; *m*-CoQA, *m*-coumaroylquinic acid; DQA, (3′,4′-dimethoxycinnamoyl)quinic acid; FQL, feruloylquinic-1,5-lactone; CQL, caffeoylquinic-1,5-lactone; *p*-CoQL, *p*-coumaroylquinic-1,5-lactone.

CGAs belong to a large group of naturally occurring compounds, synthesized in plants by the esterification of caffeic acid and quinic acid, as described in Figure 2A. Depending on the different esterification sites of quinic acid, CGAs can exist in numerous structural isomers, as shown in Figure 2B and more isomers of CGA are shown in Figure 3. 

Moreover, CGAs are important phenolic compounds in plants, commonly found in fruits, vegetables, spices, and agricultural by-products, such as potato, artichoke, chicory, apple, mulberry, carrot, eggplant, sea buckthorn and tobacco waste [11,16,45,46,47,48,49]. As shown in Table 1, there are significant differences in CGA content among different natural sources.

Coffee is considered a significant source of CGAs. In green coffee beans, the total CGA content ranges from 6 to 12% by weight [50], with the most abundant component, 5-caffeoylquinic acid (5-CQA), accounting for approximately 76–84% of total the CGAs at a concentration of about 100 mg/g.

The composition of CGAs varies depending on the plant organ from which they are derived and the extraction method used. For example, artichoke buds contain 0.503 mg/g CGA, 0.008 mg/g isochlorogenic acid B, 0.162 mg/g isochlorogenic acid A, and 0.030 mg/g isochlorogenic acid C [51]. Leaf heads contain CGA contents ranging from 49.2 mg/100 g (*Cynara algarbiensis*) to 159.5 mg/100 g (*Cynara baetica*) [52]; even solid waste retains 1901.5 ± 4.9 mg/kg CGA [53]. Furthermore, high yields of 4.95 mg/$g_{DM}$ of 5-CQA and 5.41 mg/$g_{DM}$ of 3,5-diCQA were obtained from forced chicory roots under 107 °C with 46% ethanol and 95 °C with 57% ethanol [54,55]. Additionally, when using choline chloride/1,4-butanediol (1:2) with a water content of 30% (*w*/*w*), sunflower seeds achieved the highest extraction yield of 6.16 mg/g CGA [56].

**Table 1:** CGA content in different natural sources.

Food Sources	CGA Content (mg/100 g)	Ref.
Green coffee beans	5000–12,000	[57]
Potatoes	118–13,681	[58]
Tabacco	348–2031	[8]
Artichoke	36.3–748	[52,59]
Chicory	23–644	[54]
Sunflower	214.3–616	[56]
Sea buckthorn	1.40–2010	[47]
Mulberry	259–1338	[49]
Pear	106.7–247.5	[60]
Apple	0.70–167.57	[45]

### 2.2. Bioavailability and Stability

The main functional subtypes of CGAs include caffeoylquinic acids (CQAs), diacylquinic acids (diCQAs), feruloylquinic acids (FQAs), *p*-coumarylquinic acids (*p*-CoQAs), and quinolones formed during roasting [61]. Among these, monoacylquinic acids (e.g., 5-CQA, 3-CQA, 4-CQA) exhibit relatively high bioavailability. In contrast, diacylquinic acids (e.g., 3,5-diCQA, 1,5-diCQA) show lower bioavailability. They are scarcely hydrolyzed by small-intestinal enzymes and primarily rely on colonic microbiota for degradation [62].

The clinical applications of CGAs are limited by their poor lipid solubility, permeability, and oral bioavailability. The low oral bioavailability of CGAs is attributed to their low lipophilicity, which restricts passive transmembrane transport, and the presence of an active efflux mechanism in the intestine [63], leading to inefficient intestinal absorption [64]. Scientists have conducted extensive research to address these challenges. According to Guo et al. [65], the lipophilicity of CGAs can be improved by esterification reactions using octanol under solvent-free conditions and Zhang et al. [66] found that liposomal formulations can also improve the bioavailability of CGAs after oral administration. Trivedi et al. [67] demonstrated the formulated nanophytovesicles enhanced oral bioavailability and increased transdermal permeability. For food systems, Wang et al. [68] enhanced the lipophilicity of CGA by using acyl chlorides with various degrees of unsaturation, which notably increased its antioxidant activity in both fish oil and β-carotene/linoleic acid emulsions. Karina et al. [69] also found that the short-chain esters (C4-C6) of butyl-CGA significantly enhanced rapeseed oil stability.

Interestingly, although the direct absorption of intact CGAs is limited, the overall bioavailability of their metabolites generated via intestinal absorption and microbial metabolism is quite considerable. It has been confirmed that the metabolic pathways of CGAs generally adhere to the following pattern: After ingestion, the bloodstream rapidly absorbs a portion of unchanged CGAs. Meanwhile, another portion is hydrolyzed and enzymatically converted into caffeic acid and quinic acid in the small intestine [70]. The remaining portion reaches the colon, where it is metabolized by gut microbiota to facilitate reabsorption. In plasma, the absorbed ferulic and caffeic acids and their derivatives are mainly found in conjugated forms. Finally, unabsorbed CGAs may be conjugated with sulfate or glucuronic acid, then enter peripheral circulation and undergo hepatic processing [71]. Any residual intact CGAs in the colon are hydrolyzed into primary metabolites [72]. These substances further degrade into benzoic acid, which is absorbed and ultimately excreted as hippuric acid in urine [73].

Furthermore, there are significant differences in the absorption of CGAs by different tissues [73]. Individual variations in metabolic enzyme activity, along with differences in the activity and composition of intestinal microbiota esterases, also contribute to variations in the bioavailability of CGAs. Individual differences in gut microbial ecology further contribute to heterogeneous hydrolysis capacity, making personalized responses to dietary CGAs highly variable.

As polar organic acids, CGAs exhibit low chemical stability, primarily because their molecular structure contains an *o*-diphenol hydroxyl group, which is served as an ideal substrate for phenolase catalysis and is easily oxidized under heat or light exposure [74]. Meanwhile, CGAs have high stability under acidic conditions (pH < 5.0), while a relatively low stability in alkaline environments (pH ≥ 6.0) [75]. Additionally, the presence of phenolic hydroxyl groups, unsaturated double bonds, and ester bond structures makes CGAs easily hydrolyzed and oxidized under normal or high temperatures, thereby affecting the stability of CGAs [76]. However, Luo et al. [77] found that grafting CGA onto a soluble oat β-glucan is a feasible solution to enhance its stability, promoting its application in the food industry.

### 2.3. Benefits and Side Effects

CGAs offer several health benefits, most notably a reduction in the risk of various chronic diseases such as diabetes, hypertension, and atherosclerosis. Through modulating some key signaling pathways, such as MAPK and NF-κB/NOD-like receptor protein 3 (NF-κB/NLRP3), they demonstrate strong antioxidant, anti-inflammatory, and immunomodulatory activities. In a study of their gut-protective effects, Xie et al. [78] stated that CGAs can decrease the relative abundance of *Sutterella* and *Akkermansia* while increasing the abundance of *Ruminococcus*, thereby inhibiting the development of intestinal inflammation. Moreover, CGAs can further inhibit oxidative stress and strengthen the gut barrier by activating the nuclear factor erythroid 2-related factor 2/heme oxygenase-1 (Nrf2/HO-1) pathway [79] and can prevent gastrointestinal mucosal damage in patients treated with indomethacin [80]. Ran et al. [81] demonstrated that CGAs alleviate gut aging by suppressing levels of the pro-inflammatory factors TNF-α, IFN-γ, 1L-1β and IL-6, confirming CGAs possess strong antioxidant and anti-inflammatory effects. Chen et al. [82] further found that CGAs inhibited the expression of SCD-1, reducing hepatic triglycerides, total cholesterol, and free fatty acids in non-alcoholic fatty liver disease (NAFLD). In addition, CGAs stimulate insulin secretion, enhance glucose tolerance, and improve lipid metabolism [83], which particularly highlight their anti-diabetic and anti-obesity effects [84]. Yan et al. [85] also confirmed that CGAs help recover bacterial alpha and beta diversity in the cecum and the wealth of the phylum *Bacteroidetes* and the genera *Lactobacillus*, *Blautia*, and *Enterococcus*. Furthermore, CGAs offer anti-atherosclerotic benefits by inhibiting oxidized low-density lipoprotein (LDL) formation [86] and protecting mitochondrial function via Sirtuin 1 (SIRT1) and AMP-activated protein kinase/peroxisome proliferator-activated receptor γ coactivator 1 (AMPK/PGC-1) upregulation [87]. In addition, they also can promote the repair of pancreatic tissue [88]. Specifically, CGAs prevent the activation of extracellular signal-regulated kinase 1/2 and inactivation of protein kinase B by removing excess ROS, which efficiently alleviate noninflammatory-induced diseases like Parkinson’s disease [89,90,91,92,93]. These multifaceted mechanisms support the potential of CGAs as a viable therapeutic strategy against neurodegenerative diseases.

Through the use of ex vivo and in vitro profiling assays in accordance with the ICH S7A guideline, CGAs exhibit the fundamental characteristics required of safe compounds in pharmacology safety [94]. At present, a tolerable upper intake level for CGAs cannot be established due to the insufficiency of data. Based on research from population intervention studies by Yu et al. [41], the estimated dietary intake of CGAs is 5 to 1000 mg/d, which is safe to ingest, and a daily CGA intake of ≥200 mg/d is suggested to improve fasting blood glucose. Specifically, Sascha et al. [95] estimated a human equivalent dose for a 70 kg adult is 189 mg/kg per day or 13.2 g/day. However, high-dose consumption may disrupt cellular redox homeostasis. Pang et al. [96] found that CGA concentrations exceeding 600 μM in vitro significantly inhibit hepatocyte growth, while lower concentrations of CGA (50–200 μM) play a pivotal role in protecting hepatocytes and enhancing mitochondrial energy metabolism. Moderate supplementation is advised to maximize health benefits.

## 3. Application of CGA as a Natural Antioxidant in Various Foods

Based on previous research, current application of CGAs in the food industry is primarily focused in primarily concentrated in a few specific sectors, including seafood, meat, and baked goods. CGA has shown promise in extending shelf life, inhibiting lipid oxidation and protein degradation, delaying microbial growth, and improving sensory properties. Its mechanisms of action include inhibiting endogenous lipoxygenase activity and the NF-κb inflammatory pathway, promoting the stabilization of protein networks, strengthening intestinal barrier function, and modulating the gut microbiota. These findings collectively indicate that CGA has broad application prospects in the food industry.

### 3.1. Seafood Products

CGA currently has multiple applications in the preservation of seafood products, exhibiting antioxidant, antibacterial, and immune-regulatory properties, while also enhancing the activity of lipase and amylase in fish intestines; more details are summarized in Table 2.

#### 3.1.1. Direct Addition

Direct addition of CGA, such as surface coating with powder or immersion in CGA aqueous solutions, is the most common method for using CGA in food preservation. For example, Liu et al. [97] found that in dry-cured mackerel surface coating with CGA powder (0.8% mixed with salt) inhibited lipoxygenase activity, suppressing lipid oxidation and significantly reducing the accumulation of aldehydes. Ning et al. [98] found that immersing golden pomfret filets in a 2 g/L CGA solution for 10 min reduced TVB-N to 25.90 mg N/100 g and preserved myofibrillar protein structure after 15 days, extending shelf life by 3 days, demonstrating its potential for quality maintenance.

Additionally, Li et al. [99] found that CGAs can effectively inhibit the thermal decomposition of trimethylamine oxide (TMAO) in squid extract, with the content of TMAO increased by 11.79% with added 2.5 g/L CGAs, and interestingly, Hou’s team found that the addition of 10 mg/mL CGA extracted from celery in ready-to-eat sea cucumbers can enhance their thermal stability and shelf life because CGA can promote cross-linking between molecules [100,101].

#### 3.1.2. Feed Additive

CGA also offers multifunctional benefits as a seafood feed additive. It significantly enhances growth performance, immune function, and antioxidant capacity in various fish species, with optimal dietary levels ranging from 197.5 to 1173.11 mg/kg depending on species [102,103,104,105]. CGA effectively regulates lipid metabolism, reduces lipid accumulation and protects liver health [106,107]. Additionally, when the concentration of CGA increased, the activity of catalase, glutathione peroxidase, and superoxide dismutase increased and malondialdehyde activity decreased, which improved immune-antioxidant capacity in common carp [105,108]. CGA also exhibits notable antiviral activity against pathogens like *Micropterus salmoides* rhabdovirus [109].

#### 3.1.3. Films

CGA is incorporated into films through physical incorporation or encapsulation and chemical conjugation or grafting. Increasing interest has been shown in the addition of CGA into chitosan (CS) film due to the improvement of both its functional and physical properties. For instance, Cao et al. [110] incorporated 0.2%, 0.5%, and 1.0% CGA into 2% CS coatings for snakehead fish stored at 2 °C for 5 months, finding that 0.5% and 1.0% CGA significantly inhibited lipid oxidation and delayed pH increase, but 1.0% CGA caused undesirable browning, with 0.5% recommended as optimal for sensory quality. Additionally, Hu et al. [111] prepared CGA-grafted-CS conjugated films (grafting ratios: 60, 170, 250 mg/g) and physically incorporated CGA–chitosan films for shrimp preservation, finding that the conjugated films (CGA-grafted–CS III, 250 mg/g) exhibited superior performance in maintaining shrimp quality during storage, reducing weight loss to 3.5%, total volatile base nitrogen (TVB-N) to 18.17 mg/100 g, and total bacterial count to 4.89 log CFU/g at day 8. Moreover, Yang et al. [112] grafted caffeic acid, gallic acid, and CGA onto CS (grafting ratios: 45.40, 52.70, 69.96 mg/g, respectively) for sea bass preservation. The results indicated that CGA-grafted CS exhibited the highest DPPH scavenging activity (>90%) and the strongest inhibitory effect against *Shewanella putrefaciens*, extending the shelf life of sea bass by up to 6 days. Furthermore, Chen et al. [113] developed gelatin/wheat gliadin electrospun films encapsulating 0–150 mg CGAs, showing that >100 mg CGA exhibited >90% antioxidant activity, reducing weight loss to 3.87% during 10-day storage, TVB-N to 22.87 mg/100 g, and maintaining hardness at 15.77 N. In addition, Ge et al. [114] prepared a CGA–gelatin conjugate with CGA accounting for 8.48% w/w for sword prawn preservation over 23 days, demonstrating that CGA–gelatin treatment significantly inhibited microbial growth, delayed protein degradation, inhibited lipid oxidation, and extended the shelf life from 8 to 13 days. These findings collectively reveal CGAs multifunctional role in seafood product preservation through their antioxidant and antimicrobial activities.

**Table 2:** CGA added as an antioxidant in seafood products.

CGA Addition Form	Seafood Product	Concentration of CGA	Results	Refs.
Direct addition	Mackerel (*Scomberomorus niphonius*)	0.8% CGA	CGA inhibited the effect of lipoxygenase activity on the promotion of fatty acid oxidation and key flavor accumulation in the dry-cured mackerel.	[97]
	Golden pomfret	1, 2, and 4 g/L	CGA significantly extended the shelf life of golden pomfret, especially with use at 2 g/L, and the value of the total volatile basic nitrogen (25.90 mg N/100 g) was significantly decreased after 15 d.	[98]
	Squid	2.0 mL of 1 g/L	CGA displayed strong free-radical scavenging capacity, which inhibited the thermal decomposition of trimethylamine oxide in squid extract.	[99]
	Ready-to-eat sea cucumber	1.25, 2.5, 5 and 10 mg/mL	Ready-to-eat sea cucumbers with 10 mg/mL CGA had improved thermal stability, good sensory acceptability and an extended shelf life.	[100]
	Ready-to-eat sea cucumber	5 mg/mL	CGA enhanced sea cucumber collagen stability and extended its shelf life.	[101]
Feed additive	Yellow pond turtles (*Mauremys mutica*)	100, 200, 400, 800 mg/kg	The optimal dietary CGA concentration for *M. mutica* ranges from 197.5 to 359.39 mg/kg to improve immune and antioxidative capacity.	[102]
	Rainbow trout	200, 400, 600, and 800 mg/kg diet	600–800 mg/kg CGA was recommended due to producing the best growth parameters, immune indices, and antioxidant capacity.	[103]
	Carp (*Cyprinus carpio*)	100, 200, 400, 800 mg/kg	400 mg/kg was the optimal amount of CGA addition in common carp diets, which enhanced growth performance and antioxidant capacity.	[104]
	Blackspotted Croaker *Protonibea diacanthus*	100, 200, 400, 800, and 1600 mg/kg	Catalase, glutathione peroxidase, and super oxide dismutase activity increased with the CGA concentration in the diet. A dietary CGA supplementation of 1173.11 mg/kg is suggested based on the muscle texture quality.	[105]
	Largemouth Bass (*Micropterus salmoides*)	60, 120, 180, and 240 mg/kg	Largemouth bass groups supplemented with 180 and 240 mg/kg CGA displayed improved antioxidant status and weakened inflammatory response.	[106]
	Zebrafish	\	CGA possessed significant antioxidant properties, effectively scavenging free radicals and inhibiting lipid peroxidation.	[107]
	Grass carp	400 mg/kg	A 400 mg/kg CGA supplement promoted digestibility, flesh water-holding capacity and antioxidant capacity.	[108]
	Largemouth bass (*Micropterus salmoides*)	25, 50, 100 μg/mL	CGA delayed the onset and incidence of the *Micropterus salmoides* rhabdovirus (MRSV) and reduced mortality, having been used for preventing MRSV in aquaculture farms.	[109]
	Spotted sea bass	100, 200, 300, and 400 mg/kg	Dietary CGA presented antioxidant capacity, immunity, and intestinal health benefits in a high-fat diet, and feed including 400 mg/kg CGA significantly increased total antioxidant activity.	[115]
	Juvenile common carp	550 mg/kg	The addition of both *Lactobacillus helveticus* and CGA has achieved the best outcomes in increasing catalase and glutathione peroxidase activities and decreasing malondialdehyde activity, which improved immune-antioxidant capacity in common carp.	[116]
Films				
CGA-CS	Snakehead	0.2%, 0.5%, and 1.0% (*w*/*w*) in 2% chitosan solution	The additional CGA further enhanced the antioxidant and antimicrobial properties but did not influence the hardness of snakehead fish filets after preservation.	[110]
CGA-grafted chitosan	*Penaeus vannamei*	0.440, 1.318, 2.197 g	The antioxidant and antibacterial activities of CGA-grafted–CS films increased with increasing CGA content.	[111]
CGA-grafted CS	Japanese sea bass (*Lateolabrax japonicus*)	5.0, 10.0, 20.0 mg/mL	CGA-grafted CS inhibited the growth of *Shewanella putrefaciens* and spoilage microorganisms, prevented lipid oxidation, and maintained the sensory properties of fish filets.	[112]
Gelatin/wheat gliadin electrospun films containing CGA	Grass carp	25, 50, 75, 100, 125, and 150 mg	The gelatin/wheat gliadin/CGA films delayed the oxidative deterioration of food and maintained its quality.	[113]
CGA–gelatin	Sword prawn (*Parapenaeopsis hardwickii*)	8.48% (*w*/*w*)	CGA–gelatin extended the shelf life of sword prawn from 8 days to 13 days and reduced quality loss, with the sensory evaluation appearing acceptable.	[114]

### 3.2. Meat

During processing and storage, meat products are prone to problems such as microbial contamination [117], lipid oxidation [118], and the formation of harmful substances [119]. These problems not only shorten the product shelf life but may also affect food safety. As a natural phenolic compound widely present in plants, CGA possesses excellent antibacterial and antioxidant activity. Therefore, it has been extensively studied to address the quality and safety challenges of meat products, with relevant research findings as follows in Table 3.

Research indicates that in terms of microbial control and preservation, a 3 log cfu/g reduction in the viable count of *Salmonella enteritidis* S1 and an extension of the microbiological shelf life by 3–6 days were achieved by treatment with 2 mM CGA [120]. The growth of *Yersinia enterocolitica* in raw pork also is inhibited by CGA used as a natural antibacterial preservative [121].

Moreover, Ding et al. [122] demonstrated that CGA, through its antioxidant properties, significantly inhibited the formation of heterocyclic amines in roasted lamb at an optimal addition level of 0.125 mmol/100 g by competitively consuming key precursors such as glucose and amino acids. Additionally, Bergamaschi et al. [123] found that the CGA from the primary bioactive constituent of defatted green coffee bean extract inhibited lipid oxidation in cooked pork burgers, reducing thiobarbituric acid reactive substances (TBARS) values from 1.28 to 0.47 mg malondialdehyde/kg (MDA/kg) and extending the shelf life, while concomitantly preserving color stability and exhibiting no adverse sensory effects at an optimal concentration of 0.15%. Interestingly, through enhancing antioxidant capacity and regulating the phosphorylation modification of key proteins, CGA significantly improved the meat quality of immunologically stressed broilers [124].

Furthermore, CGA exhibits synergistic effects with other substances in preserving meat freshness. For example, Yang et al. [125] found the synergistic treatment of CGA with cold plasma yielded optimal results at 15 min, which significantly improved the color (redness increased by 40.7%), tenderness (shear force reduced by 34.09%), and flavor of roasted mutton patties, and inhibited lipid oxidation, decreasing the TBARSs by 58.78%. In addition, Yang et al. [126] stated that in an edible coating, the synergistic combination of CGA—the predominant phenolic compound in *Aronia melanocarpa* pomace extract—and gelatin significantly inhibited lipid oxidation and microbial growth in chilled pork, which extended the shelf life from 9 to 12 days, delaying protein decomposition and drip loss and preserving meat color, texture, and sensory quality. Meanwhile, Zou et al. [127] illustrated CGA incorporated into a gelatin–chitosan–glycerol edible coating significantly improved the color stability and the inhibited lipid oxidation and microbial growth of fresh beef, extending the shelf life by 3–6 days.

### 3.3. Baked Food

Given the rich content of CGA in natural plant-derived materials, these sources have been widely investigated in studies for incorporation into baked products, aiming to boost their nutritional properties and antioxidant capacity.

The incorporation of coffee-derived CGA through green coffee bean (GCB) powder, roasted coffee bean extract, or coffee leaf powder, either as a dry powder or as an aqueous extract blended into wheat flour, notably increased the total phenolic content (TPC) and antioxidant capacity of bread or rusk. CGA also exhibited good thermal stability during baking, and in some formulations, antioxidant activity was further enhanced after the baking process. Zain et al. [128] found that bread with 3% GCB added increased TPC from 0.26 mg gallic acid equivalent/g (GAE/g) to 0.93 mg GAE/g, and the DPPH free-radical scavenging ability (IC50) decreased from 11.25 mg/mL to 3.28 mg/mL, while the iron ion-chelating ability increased from 0.02 mg ethylenediaminetetraacetic acid/g (EDTA/g) to 0.44 mg EDTA/g. Meanwhile, Silva et al. [129] explained that when an aqueous extract of roasted coffee beans was used to replace water in bread formulations, the 50% substitution was found to achieve the optimal balance between antioxidant capacity and sensory acceptability. Also, Patil et al. [130] found that the TPC of the sample with 3% coffee leaf powder added increased from 168.22 mg/100 g to 292.52 mg/100 g, and the 2, 2′-Azino-bis (3-ethylbenzothiazoline-6-sulfonic acid, ABTS) antioxidant activity rose from 697.9 μM Trolox equivalent antioxidant capacity/g (μMTEAC/g) to 1213.18 μMTEAC/g. Furthermore, Anthony et al. [131] reported that gingerbread enriched with 10% GCB (mainly CGA) showed a significant rise in both TPC and antioxidant capacity. Additionally, when added within the recommended range, the texture, chewiness, and springiness compared to the control samples displayed no significant differences.

At the same time, plant tissues rich in CGA, such as sweet potato leaf powder [132], artichoke leaves [133], and defatted sunflower seeds [134], play an important role in elevating TPC and antioxidant capacity, as well as potentially lowering the glycemic index. For example, Leonardo et al. [134] found that defatted sunflower seed flour was used in breads to enhance protein content and increase antioxidant activity (995 ${\mu mol}_{Trolox}$·${g_{bread}}^{-1}$, Trolox equivalent antioxidant capacity per gram of bread) and showed significant α-amylase inhibition of 92.81% and pancreatic lipase inhibition of 25.6%. 

### 3.4. Others

CGA has also been demonstrated to play a versatile role in enhancing the functional and nutritional properties of various food systems.

Gastélum-Estradaand et al. [135] demonstrated that wounding stress technology increased the CGA content in carrots by 83%. When combined with orange juice and broccoli sprouts to formulate a functional beverage, the product exhibited a significant enhancement in antioxidant activity (+26%), along with promising anti-inflammatory and anti-obesity potential. The beverage maintained good stability after 28 days of storage at 4 °C, offering a feasible strategy for the development of natural and healthy functional fruit and vegetable beverages. In fruit-enriched wheat beers, CGA was the predominant phenolic acid, reaching its highest levels in beers with Kamchatka berries, and was also noted for its antioxidant activity, DNA protection, and contribution to the fruit aroma [136]. Interestingly, Wu et al. [137] found that six phenolic compounds, including CGA, were identified as the core antioxidant components in sugarcane vinegar, synergistically enhancing antioxidant activity in an acidic environment and modulating oxidative stress through multi-target regulation. Furthermore, the addition of 0.5–0.75% CGA derived from green coffee extract to hazelnut paste significantly extended its shelf life from 28 to 60–90 days, inhibited lipid oxidation, and protected endogenous tocopherols, offering a viable strategy for the natural preservation of high-fat foods [138].

In addition, by subjecting lotus seed starch to high-pressure sterilization in the presence of CGA, Wang et al. [139] found that the resulting complex achieved a resistant starch content of 63.85%, effectively inhibiting starch retrogradation and reducing digestibility, offering a theoretical foundation for the development of low-glycemic-index functional starch-based foods.

## 4. Encapsulation Strategies for CGA in Food Matrices

The poor stability and bioaccessibility of CGA present significant challenges to its application in foods. Therefore, encapsulation technologies have been widely adopted to enhance the stability, specificity and bioavailability of bioactive substances. Consequently, the encapsulation strategies applied to CGA can be broadly classified into liposomes, nanoparticles, microcapsules, hydrogels and emulsions [140].

Fu et al. [141] synthesized supramolecular traditional Chinese medicine nanoparticles using berberine and CGA self-assembly, which played a role in inhibiting the growth of *Staphylococcus aureus*, a common foodborne pathogen, in food matrices. Notably, in 2023, Zhang et al. [142] developed a ferritin CGA-loaded sodium alginate (SA)–apoferritin(Apo) complex (SA-Apo-CGA) using the unique hollow spherical structure of ferritin; a promising nanocarrier for functional food substances. The retention rate of CGA was 96.90%. Subsequently, in 2025, the same team [143] optimized the ultrasound-assisted extraction process, achieving an extraction yield of 139.46 mg/kg, which stood out for its excellent biostability in food industry.

According to Ibrahim et al. [144], microencapsulation can solve the problems of thermal-sensitive and easily oxidized CGA. They used gum Arabic and maltodextrin to encapsulate CGA from green coffee bean extract by spray drying. The result showed a high encapsulation efficiency of 98.48% and 99.63% for 200 mg powder, respectively. At the same time, Sandra et al. [145] found that using 3% fructan and 2% gum Arabic as wall materials in the spray-drying process provided an encapsulation efficiency of 94.36%, while retaining a CGA concentration of 27.45%. Notably, Paim et al. [146] found that utilizing zein as a carrier for CGA in spray drying achieved an encapsulation efficiency of 75.2% and enhanced thermal stability by 40 °C. These commonly used wall materials have been widely applied in the food industry, making the application of CGA more practical.

Meanwhile, Paredes-Toledo et al. [147] reported that the co-encapsulation of CGA and curcumin in a spray-dried linseed oil multiple emulsion increased the bioaccessibility (47.6%) and retention rate of CGA, supporting its potential as multifunctional delivery systems for functional foods. Additionally, Pan et al. [148] discovered a promising fruit preservation hydrogel loaded with CGA–zinc nanoparticles, which played a significant role in reducing fruit respiration rates and inhibiting the deterioration and spoilage of fruits. The inhibition rates of this hydrogel against *Staphylococcus aureus* and *Escherichia coli* were 95.30 ± 0.53% and 95.27 ± 1.80%, respectively.

In summary, encapsulation strategies are indispensable for addressing the issues of CGA’s high sensitivity, poor water solubility and low bioavailability, as well as for improving its thermal stability, oxidation resistance, and controlled release capacity. Future research should focus on expanding these technologies to broaden their benefits.

## 5. Sensory Evaluation of Food Products Containing CGA

It is crucial to deeply explore customers’ preferences to evaluate food products and gain market success among diverse age profiles through the sensory evaluation of food [149]. Although some studies have examined the flavor, texture and overall customer acceptance of meat products, baked goods, and so on, there is still a dearth of studies focusing on the sensory characteristics of CGA in the broader food industry.

Researchers mainly focus on the influence of adding CGA on the meat’s organoleptic properties, such as the color, texture, aroma, juiciness, and tenderness. Yang et al. [125] observed that cold plasma could significantly improve the texture and color profile of the muscle protein, so they aimed to investigate the influence of synergistic treatment of CGA with cold plasma on roasted meat quality. The results show that after 15 min of treatment with CGA and cold plasma, the samples achieved the highest scores for flavor (4.73/5) and overall acceptability (4.68/5), as well as a markedly lower shear force (13.9 N compared to 21.09 N for control), which was determined by the inhibition of fat oxidation and the capability to scavenge free radicals. Notably, the concentration of hexanal dropped from 1082.36 μg/kg in the control to 469.95 μg/kg, and the levels of other oxidation products, including furans, esters, ketones, alcohols, were also reduced, helping to preserve freshness and minimize off-flavors. Moreover, the addition of CGA to other meats also has a similar effect. In an experiment concerning the improvement of a gelatin–chitosan–glycerol edible coating, it was found that on the 12th day of storage the addition of CGA provided the least reduction in chewability, better protection of the color, and the highest improvement effect on the preservation of beef [127]. Furthermore, the addition of defatted green coffee beans, which are rich in CGA, had a very low impact on the appearance, color, and aroma of raw and cooked pork hamburgers, and even had only a slight impact after high-concentration addition [123].

In baked products, the addition of CGA results in more obvious bitterness and color changes, but significantly improves the chewiness, texture, hardness, aroma, and other aspects. Zain et al. [128] found the addition of CGA caused the surface of the green coffee bean (GCB) bread to take on a slightly greenish color, resulting in a minor negative impact on the color score, changing from 5.73 to 5.27–5.00/9 points. The odor scores significantly dropped from 5.20 points in the control group to 3.90–4.30/9 points, and the taste score decreased from 5.30 points to 4.17–4.27/9 points. This was speculated to be related to 2,4,6-trichlorophenol potentially generated by CGA, a compound primarily responsible for stale or musty flavors. Bread containing 3% GCB was accepted by the majority of people. Meanwhile, the CGA present in the added coffee leaf powder was the main cause of darkening of color and the intensification of bitterness in wheat flour rusk [130]. Furthermore, Silva et al. [129] stated that these changes in color, bitterness, and coffee flavor caused by CGA could be sold as a distinctive product, mainly targeting bitter foods and coffee lovers, which effectively addresses the impact of these changes.

Based on the above, although the addition of CGA can effectively enhance the acceptance and storage time of food, it often comes with potential negative impacts on the color and flavor of the products. In the future, a more in-depth analysis of the interaction mechanism between CGA and food substrates, as well as consumer-oriented acceptance research, will be the key to driving its more precise and wider application in the food industry.

## 6. Conclusions

CGAs are widely present in fruits, vegetables, and food by-products. Due to their potent antioxidant, anti-inflammatory, and immunomodulatory activities, CGAs also offer a range of health-promoting benefits, including protection of the gastrointestinal, cardiovascular, and nervous systems. Furthermore, CGAs offer numerous advantages in food preservation. They can effectively inhibit lipid oxidation and microbial growth, extend shelf life, and enhance the storage stability and safety of seafood products, meat, and baked foods, laying the foundation for further expansion of their application in a diverse range of food products.

However, several challenges limit the practical application of CGAs in the food industry. First, there is a lack of empirical research on the influence of adding CGAs to foods on their sensory characteristics, particularly in the case of seafood. Although existing studies have shown that CGAs can improve antioxidant capacity, suppress the formation of undesirable flavor compounds, and improve texture, such as increasing the tenderness of meat and the chewiness of bread, they may also impart bitterness or alter color. Therefore, ensuring that CGAs do not negatively impact consumer acceptance is an area worthy of further exploration. Second, their chemical structure makes them susceptible to oxidative degradation under heat, light, or alkaline conditions, which limits their large-scale application in actual food systems. Furthermore, their low lipid solubility and poor permeability result in low oral bioavailability. Current cutting-edge research is addressing this gap by developing encapsulation strategies such as microcapsules, nanocapsules, and hydrogels. Unfortunately, due to the additional expenses, need for specialized equipment, and potential impacts on consumer acceptance, only a few studies have actually applied these innovations to food products. More efficient and cost-effective methods should be developed. Finally, CGAs are typically incorporated into foods either by dissolving them in solvents or adding them to feed additives, reflecting the relatively narrow range of their application methods. Therefore, the development of more practical and diverse forms of CGAs as food additives will become a major trend in the future, so that multifaceted health benefits and dietary improvements can be achieved.

## Figures and Tables

**Table 3:** CGA added as an antioxidant in meat products.

Food Product	Concentration of CGA	Results	Refs.
Chilled fresh chicken	2 mM (the minimum inhibitory concentration, MIC)	A 3 log cfu/g reduction in the viable count of *Salmonella enteritidis* S1 and extension of the microbiological shelf life by 3–6 days were achieved by treatment with 2 mM CGA.	[120]
Raw pork	2.5 mg/mL (MIC)	As a natural antibacterial preservative, CGA can inhibit the growth of *Yersinia enterocolitica* in raw pork and *Enterobacter sakazakii* in skim milk.	[121]
Charcoal-roasted lamb meats	0.025, 0.125, and 0.625 mmol	The generation of heterocyclic amines in charcoal-roasted lamb patties was significantly inhibited by the addition of 0.125–0.625 mmol/100 g CGA and epicatechin.	[122]
Raw and cooked pork meat burgers	183.1 ± 19.3 mg/g	dGCB extract (containing CGA) is an ideal natural antioxidant for the shelf life of vacuum-packed and refrigerated burgers.	[123]
Broiler meat	500, 750 (mg/kg)/day	CGA increased antioxidant enzymatic activity and decreased the malonaldehyde and protein carbonyl level in broiler breast meat.	[124]
Roasted mutton patties	2 mmol/L	A synergistic treatment of CGA and cold plasma results in a long-lasting red color and enhanced tenderness of the roasted meat and significantly improves free-radical scavenging capacity, inhibiting lipid oxidation by 39.19–64.84%.	[125]
Chilled pork	36.91 mg/100 mg	Gelatin edible coatings containing *Aronia melanocarpa* pomace polyphenols inhibited TVB-N in chilled pork, which demonstrates that the G/AMPPs coating significantly extended the shelf life of chilled pork by up to 15 days.	[126]
Fresh beef	0.5% *w*/*w* (gelatin–chitosan–glycerol edible coating)	CGA delayed the increase in the total volatile base nitrogen and inhibited the growth of microorganisms on fresh beef.	[127]

## Data Availability

No new data were created or analyzed in this study. Data sharing is not applicable to this article.

## Abbreviations

The following abbreviations are used in this manuscript:

(3′,4′-dimethoxycinnamoyl)quinic aid	DQA
1,4-dicafeoylquinic acid	1,4-diCQA
1,5-dicafeoylquinic acid	1,5-diCQA
1-*O*-caffeoylquinic acid	1-CQA
1-*O*-feruloylquinic acid	1-FQA
2, 2′-Azino-bis, 3-ethylbenzothiazoline-6-sulfonic acid	ABTS
2,2-Diphenyl-1-picrylhydrazyl	DPPH
3,4-dicafeoylquinic acid, isochlorogenic acid B	3,4-diCQA
3,5-dicafeoylquinic acid, isochlorogenic acid A	1,3-diCQA
3-*O*-caffeoylquinic acid, neochlorogenic acid	3-CQA
3-*O*-feruloylquinic acid	3-FQA
4,5-dicafeoylquinic acid, isochlorogenic acid C	4,5-diCQA
4-*O*-caffeoylquinic acid, cryptochlorogenic acid	4-CQA
4-*O*-feruloylquinic acid	4-FQA
5-*O*-caffeoylquinic acid, neochlorogenic acid	5-CQA
5-*O*-feruloylquinic acid	5-FQA
AMP-activated protein kinase/peroxisome proliferator-activated receptor γ coactivator 1	AMPK/PGC-1
Apoferritin	Apo
Caffeoylferuloylquinic acids	CFQA
Caffeoylquinic acids	CQA
Caffeoylquinic-1,5-lactone	CQL
Chitosan	CS
Chlorogenic acids	CGA
Colony-forming units	CFUs
Defatted green coffee bean	dGCB
Diacylquinic acids	diCQA
Ethylenediaminetetraacetic aicd	EDTA
Feruloylquinic acids	FQA
Feruloylquinic-1,5-lactone	FQL
Gallic acid equivalent	GAE
Gelatin edible coatings containing *Aronia melanocarpa* pomace polyphenols	G/AMPPs
Green coffee bean	GCB
Low-density lipoprotein	LDL
Malondialdehyde	MDA
*m*-coumaroylquinic acid	*m*-CoQA
Minimum inhibitory concentration	MIC
Mitogen-activated protein kinase	MAPK
NF-E2-related factor 2	Nrf2
Non-alcoholic fatty liver disease	NAFLD
Nuclear factor erythroid 2-related factor 2/heme oxygenase-1	Nrf2/HO-1
Nuclear factor kappa-B	NF-κB
Nuclear factor kappa-B/NOD-like receptor protein 3	NF-κB/NLRP3
*p*-coumaroylquinic acid	*p*-CoQA
*p*-coumaroylquinic-1,5-lactone	*p*-CoQL
Reactive oxygen species	ROS
Sirtuin 1	SIRT1
Sodium alginate	SA
The European Food Safety Authority	EFSA
Thiobarbituric acid reactive substances	TBARSs
Total phenolic content	TPC
Total volatile base nitrogen	TVB-N
Trimethylamine oxide	TMAO
Trolox equivalent antioxidant capacity	μMTEAC
Ultraviolet-A light	UVA
